# Activation of cannabinoid receptor type 2 attenuates surgery-induced cognitive impairment in mice through anti-inflammatory activity

**DOI:** 10.1186/s12974-017-0913-7

**Published:** 2017-07-19

**Authors:** Lingling Sun, Rui Dong, Xin Xu, Xi Yang, Mian Peng

**Affiliations:** Department of Anesthesiology, Zhongnan Hospital of Wuhan University, 169, Donghu Road, Wuhan, 430071 Hubei China

**Keywords:** Cannabinoid receptor type 2, Postoperative cognitive dysfunction, Neuroinflammation

## Abstract

**Background:**

Neuroinflammation plays a major role in postoperative cognitive dysfunction (POCD). Accumulated evidence indicates that cannabinoid receptor type 2 (CB2R) can mediate anti-inflammatory and immunomodulatory effects in part by controlling microglial activity. However, the impact of CB2R on postoperative cognition has not been investigated. We hypothesized that CB2R is involved in surgery-induced cognitive impairment in adult mice.

**Methods:**

Adult C57BL/6 mice were subjected to intramedullary fixation surgery for tibial fracture under isoflurane anesthesia and CB2R agonist (JWH133) or CB2R antagonist (AM630) treatment. The mice were trained 24 h prior to surgery using a fear conditioning protocol and assessed in a novel context on postoperative days 1, 3, and 7 to evaluate cognitive function. Open-field testing was performed to evaluate the locomotor activity of the mice. The expression levels of IL-1β, TNF-α, MCP-1, and CB2R in the hippocampus and prefrontal cortex were assessed by Western blotting; the expression of microglial marker CD11b in the CA1 area of the hippocampus and medial prefrontal cortex was assessed by immunostaining.

**Results:**

The mice displayed no changes in locomotor activity after surgery and drug treatments. The mice exhibited impaired hippocampal-dependent memory accompanied by an increased expression of proinflammatory factors in the hippocampus and prefrontal cortex 1, 3, and 7 days after surgery, while hippocampal-independent memory remained unaffected at the same time points. JWH133 treatment attenuated surgery-induced memory loss, while AM630 treatment aggravated surgery-induced memory loss, paralleled by a decreased or increased expression of proinflammatory factors in the hippocampus and prefrontal cortex. The expression of CB2R in the hippocampus and prefrontal cortex was upregulated following surgery; however, it was downregulated by postoperative treatment with JWH133. Similarly, the expression of CD11b in the CA1 area of the hippocampus and medial prefrontal cortex was upregulated following surgery and downregulated by postoperative treatment with JWH133.

**Conclusions:**

These findings indicate that CB2R may modulate the neuroinflammatory and cognitive impairment in a mouse model of orthopedic surgery, and the activation of CB2R may effectively ameliorate the hippocampal-dependent memory loss of mice in the early postoperative stage.

**Electronic supplementary material:**

The online version of this article (doi:10.1186/s12974-017-0913-7) contains supplementary material, which is available to authorized users.

## Background

Postoperative cognitive dysfunction (POCD) is increasingly recognized as a common complication of major surgery. POCD refers to a relative decline in performance from preoperative levels on neuropsychological tests of learning, memory, and executive functioning in the short and long terms after surgery [1]. Clinical studies have shown that POCD is associated with disadvantageous outcomes, including the prolongation of hospitalization, impairment in daily functioning, premature departure from the workforce, and increased risk of mortality [2]. The incidence of POCD is expected to rise, as older age has been identified as the main predisposing factor, and the population of older surgical patients is increasing [1]. However, the neuropathogenesis of POCD is still largely unknown.

An accumulating body of evidence suggests a pivotal role for inflammatory processes due to surgical trauma in the initiation of POCD [3]. Preclinical studies have also indicated that the postoperative memory deficit is paralleled by increased levels of cytokines in the plasma and hippocampus, and interrupting the inflammatory process has been found to mitigate memory dysfunction [4–6]. Under normal quiescent conditions, the immune system is activated by environmental and psychological stimuli; it secretes low levels of proinflammatory cytokines, such as interleukin (IL)-1, IL-6, and tumor necrosis factor (TNF)-α, and inflammatory mediators, such as prostaglandins; it positively regulates the remodeling of neural circuits, promoting memory consolidation, long-term potentiation (LTP), and neurogenesis. However, in conditions in which the immune system is strongly activated, such as infection or surgical trauma, glia and other brain immune cells change their morphology and function and secrete high levels of proinflammatory cytokines and prostaglandins. The overproduction of these inflammatory mediators creates a neurotoxic response; produces direct detrimental effects on memory, neural plasticity, and neurogenesis; and eventually leads to POCD [7].

Found primarily in the peripheral immune system, the cannabinoid receptor type 2 (CB2R) has anti-inflammatory and immunomodulatory actions [8]. It is now accepted that CB2R is also present in limited numbers in the central nervous system; moreover, a prominent upregulation of CB2R expression under neuroinflammatory conditions has been observed in the brain, particularly in microglia [9]. Neuroinflammation is commonly thought to be involved in the pathogenesis and progression of neurodegenerative diseases such as Alzheimer’s disease (AD), Parkinson’s disease (PD), multiple sclerosis (MS), and amyotrophic lateral sclerosis (ALS). Additionally, an elevated expression of CB2R in brain tissues has been reported in these neurodegenerative diseases [10–13]. The neuroprotective effects of CB2R activation have been confirmed in animal models, particularly in animal models for AD and stroke [14–17]. In addition, CB2R appears to play an important role in controlling chemokine-induced chemotaxis of immune cells at lesion sites [18]. Importantly, the activation of CB2R is believed to dampen the production of inflammatory mediators and to facilitate the production of prosurvival factors by controlling glial activity and toxicity [19].

Taken together, evidence casts light on a plausible link between neuroinflammation and postoperative cognitive dysfunction. There is a growing appreciation of the potential role of CB2R in immunomodulation and neuroinflammation. However, there is scarce evidence available on how CB2R contributes to neuroinflammation and cognitive function in the setting of surgery.

In recent years, the effects of the endocannabinoid system on cognition and emotion mediated by the cannabinoid receptor type 1 (CB1R) have been widely investigated, and studies have gradually begun to examine CB2R [20]. In this study, we assessed changes in the expression of CB2R and proinflammatory factors, including IL-1β, TNF-α, and monocyte chemoattractant protein (MCP)-1, in the hippocampus and prefrontal cortex in a mouse model of orthopedic surgery. We also investigated whether a selective CB2R agonist and antagonist could change the CB2R expression, neuroinflammation, and impaired learning and memory in these mice. Our primary hypothesis is that CB2R expression is associated with the neuroinflammation and cognitive impairment in mice following surgery.

## Methods

### Animals

The experimental protocol was approved by the Animal Ethics Committee of Zhongnan Hospital of Wuhan University, Hubei, China, and all experiments were performed in accordance with the National Institutes of Health Guidelines for the Care and Use of Laboratory Animals. Adult male C57BL/6 mice (4 months old, weighing 20–30 g, from the Experimental Animal Center of Hubei province) were housed five per cage with free access to food and water. The temperature, humidity, and day-night cycle were maintained according to the standards established by the experimental animal laboratory at Zhongnan Hospital of Wuhan University. The mice were acclimatized to the laboratory environment for at least 7 days prior to the initiation of experiments.

### Drugs

The CB2R agonist JWH133 and CB2R antagonist AM630 were purchased from Cayman Chemical (Ann Arbor, USA) and dissolved in DMSO, Tween 80, and distilled water (1:1:8) on each day of drug administration. The drug administration time, doses, and vehicles were chosen based on our preliminary experiments as well as previous findings [21–23]: 2 mg/kg for JWH133 and 3 mg/kg for AM630, administered intraperitoneally at a volume of 10 ml/kg. The drug treatment was initiated immediately after recovery from anesthesia and repeated every 24 h postoperatively, and behavioral tests were carried out 30 min after drug treatment.

### Experimental protocol

The mice were randomly allocated to the control group, JWH133 group, AM630 group, anesthesia group (isoflurane anesthesia), surgery group (surgery under isoflurane anesthesia), surgery + JWH133 group (JWH133 treatment after surgery under isoflurane anesthesia), or surgery + AM630 group (AM630 treatment after surgery under isoflurane anesthesia). The mice were trained for fear conditioning 24 h prior to surgery treatments. Fear conditioning tests were performed 1, 3, and 7 days after surgery. Within each treatment group, separate cohorts were subjected to assessment at each time point (*n* = 6 per cohort). The animals were sacrificed 2 h after the behavioral assessment 1, 3, and 7 days after surgery; half (*n* = 3) were used for Western blotting and half (*n* = 3) for immunostaining. In addition, the open-field test was carried out to evaluate the locomotor activity of the mice.

### Surgical model

The mice were subjected to an intramedullary fixation surgery for tibial fracture under isoflurane anesthesia as described in a previous study [24], with modifications to the anesthesia protocol. In brief, anesthesia consisted of induction with 3.0% isoflurane followed by maintenance with 1.5% isoflurane carried by 100% oxygen. A skin incision was made below the knee, the tibia was exposed, and a 0.3-mm pin was inserted into its medullary cavity, thus achieving intramedullary fixation. Next, the bone was fractured at the midpoint. Lastly, the wound was sutured after necessary debridement. The process of surgery lasted approximately 10 min, and the total duration of anesthesia was fixed at 20 min. The rectal temperature of the mice was maintained at 37 ± 0.5 °C during the surgery by controlling the temperature in the anesthetizing chamber. The mice were left to recover on heated pads after anesthesia. Following recovery, they were returned to their home cage. To treat the pain associated with the skin incision, a 2% lidocaine solution was applied locally before the incision, and 1% tetracaine hydrochloride mucilage was applied to the wound twice daily until day 3 post surgery.

### Behavioral tests

#### Open-field test

The open-field test was carried out 15 min before the training phase and each test phase of fear conditioning. Each mouse was gently introduced into the center of an open-field chamber under dim light and allowed to move freely for 5 min. The activities were automatically recorded by a video camera connected to the Any-Maze animal tracking system software (Xinruan Information Technology Co. Ltd., Shanghai, China), and movement parameters were calculated by the software. The total distance traveled was used to determine the locomotor activity of the mice. The floor of the chamber was cleaned with 75% ethanol to avoid the presence of olfactory cues after each test session.

#### Fear conditioning test

A previously published paradigm of the fear conditioning test (FCT) was adopted. This test consists of a training phase prior to surgery and a test phase 1, 3, and 7 days after surgery [24].

One day prior to surgery, the mice were trained for fear conditioning to establish long-term memory. Each mouse was placed into the conditioning chamber for a 120-s accommodation period, followed by six pairs of conditional-unconditional stimuli and another 60 s of remaining in the conditioning chamber. One pair of conditional-unconditional stimuli consists of a 20-s, 70-dB sine wave tone (conditional stimulus), then a contextual interval of 25 s (trace interval), and finally a 2-s, 0.70-mA electrical footshock (unconditional stimulus). The pairs of conditional-unconditional stimuli were separated by random intervals from 45 to 60 s.

The test phase of the FCT consists of a context test, which reflects hippocampal-dependent memory, and a tone test, which assesses hippocampal-independent memory. In the context test, the mice were simply placed back into the conditioning chamber for 5 min without a tone or footshock. The tone test was performed 2 h after the context test. The mice were placed in a novel chamber with a different shape for 5 min, during which the tone was delivered for 3 min. The percentage freezing time (defined as the time in which mice had no movements except for respiration) was recorded by the Any-Maze animal tracking system software.

### Western blotting for proinflammatory factors and CB2R

The mice in the control, surgery, surgery + JWH133, and surgery + AM630 groups were sacrificed 1, 3, and 7 days after anesthesia or surgery to assess the expression of CB2R, IL-1β, TNF-α, and MCP-1 in the hippocampus and prefrontal cortex using Western blotting. The brain tissues (hippocampus and prefrontal cortex) of the mice were harvested following decapitation. Each sample was homogenized in ice-cold lysis buffer (150 mM NaCl, 1 mM EDTA, 50 mM Tris, 1% Triton, 0.1% sodium dodecyl sulfate, and 0.5% deoxycholate) plus a protease inhibitor. The lysate was centrifuged at 12,000 rpm for 5 min at 4 °C to remove the sediment. The total protein concentrations in the supernatants were determined using a BCA protein assay kit (Aspen, Wuhan, China). The protein extracts were separated using sodium dodecyl sulfate-polyacrylamide gel electrophoresis gels and blotted to nitrocellulose membranes. The membranes were then blocked with 5% nonfat milk for 1 h at room temperature. The blots were incubated overnight at 4 °C with the following primary antibodies: anti-CB2R (1:1000, Abcam, Cambridge, UK), anti-IL-1β (1:1000, Santa Cruz Biotechnology, Inc., Santa Cruz, USA), anti-TNF-α (1:1000, Abcam, Cambridge, UK), anti-MCP-1 (1:1000, Abcam, Cambridge, UK), or anti-GAPDH (1:10,000, Abcam, Cambridge, UK). After rinsing, the membranes were incubated with horseradish peroxidase-conjugated goat anti-mouse IgG secondary antibody (Kirkegaard & Perry Laboratories, Inc., Gaithersburg, USA) for 30 min at room temperature. The specific immunoreactivity was detected using enhanced chemiluminescence (Aspen, Wuhan, China). The bands were scanned, and the optical density was measured using image analysis software (AlphaEaseFC software). The immunoreactivity of CB2R, IL-1β, TNF-α, and MCP-1 was normalized to that of GAPDH.

### Immunofluorescence for CD11b

The mice from the control, surgery, surgery + JWH133, and surgery + AM630 groups were sacrificed 1, 3, and 7 days after anesthesia or surgery treatment to assess the expression of microglial marker CD11b in the CA1 area of the hippocampus and the medial prefrontal cortex using immunofluorescence. The mice were anesthetized with isoflurane and perfused transcardially with ice-cold saline followed by 4% formalin. The fixed brain was rapidly dissected, postfixed overnight at 4 °C, embedded, and cut into serial sections containing the hippocampal CA1 area and medial prefrontal cortex. The sections were blocked with 5% bovine serum albumin for 20 min, washed in PBS, and then incubated with a mouse monoclonal antibody against the microglial marker CD11b (1:200, Abcam, Cambridge, UK) at 4 °C overnight. Next, after washing, the sections were incubated with FITC-labeled goat anti-mouse IgG (1:50, Aspen, Wuhan, China) secondary antibody at room temperature for 2 h in the dark. Lastly, after washing, the immunostained sections were visualized using a fluorescence microscope (Olympus, Tokyo, Japan) equipped with an imaging system.

### Statistical analysis

The normality of data was analyzed by the Shapiro–Wilk test, and we found that the data were normally distributed. The data are expressed as the mean ± standard error of the mean (SEM). Both the behavioral data and immunohistochemical data were analyzed using a repeated measures analysis of variance (ANOVA), followed by a Student–Newman–Keuls multiple range test for post hoc comparisons. All analyses were performed using a statistical package (GraphPad Prism 5, GraphPad, San Diego, CA, or SPSS 19.0, IBM, Armonk, NY). A *p* value less than 0.05 was considered statistically significant.

## Results

### Locomotor activity

**Fig. 1:**
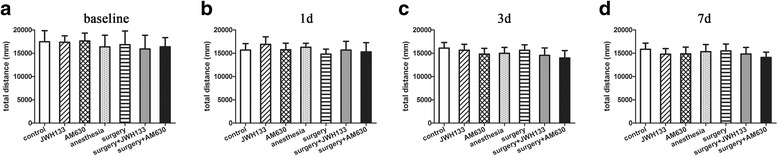
The locomotor activity of the mice was not affected by the treatments. The open-field test was performed to assess the total distance traveled 1 day before surgery (**a**) and 1, 3, and 7 days after surgery (**b**–**d**). The data are plotted as the mean ± standard error of the mean for each group (*n* = 6 per group)

We initiated the study by assessing the baseline locomotor activity of the mice 15 min before the training phase of the FCT. The effects of anesthesia, surgery, and postoperative drug treatments on locomotor activity were evaluated 15 min before each test phase of the FCT on 1, 3, and 7 days after surgery. The effect of each drug was also assessed in mice that did not undergo surgery. The total distance traveled in the open-field chamber during 5 min of exploration was used to assess the locomotor activity. A repeated measures ANOVA for the data reported in Fig. 1 identified no significant differences among the groups (Fig. 1a-d; overall *F* (6,35) = 0.228, *n* = 42, *p* = 0.965). These data indicate that the baseline locomotor activity was equal among the groups of mice, and locomotor activity was not affected by the above treatments.

### Fear conditioning test

The freezing time in the training phase of fear conditioning showed no significant differences between the groups (data not shown, Additional file 1: Figure 1), indicating that the baseline learning and memory abilities of the groups were equal.

**Fig. 2:**
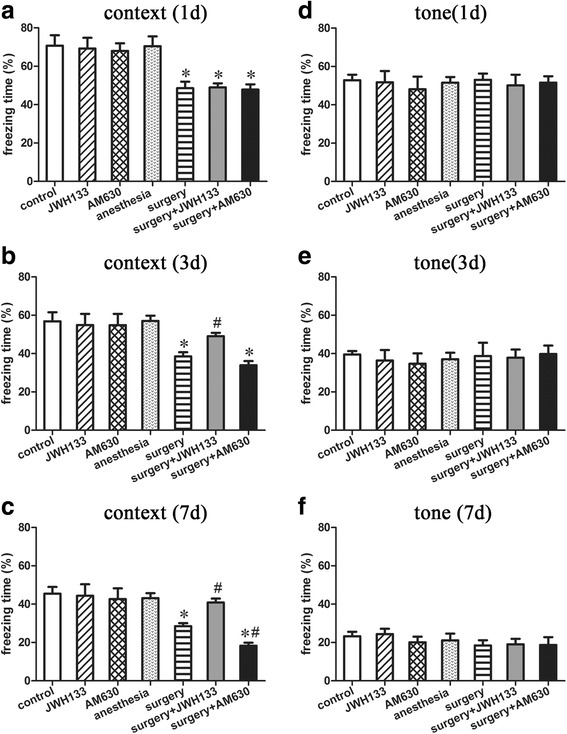
Surgery impaired hippocampal-dependent memory, but not hippocampal-independent memory. JWH133 treatment alleviated postoperative hippocampal-dependent memory loss, while AM630 treatment aggravated it. The percent freezing time in the context test (**a**–**c**) and tone test (**d**–**f**) were assessed 1, 3, and 7 days after surgery. The data are plotted as the mean ± standard error of the mean for each group (*n* = 6 per group). *p < 0.05 versus the control group, ^#^ *p* < 0.05 versus the surgery group

The context test of the FCT was performed to assess hippocampal-dependent memory. A repeated measures ANOVA for the data reported in Fig. 2 identified differences among the groups (Fig. 2a–c; overall *F* (6,35) = 15.036, *n* = 42, *p* < 0.05). Compared to the control condition, treatment with JWH133, AM630, or isoflurane anesthesia did not significantly change the freezing time of mice in the context test at any time point (Fig. 2a–c; *p* > 0.05 for each). Surgery under isoflurane anesthesia decreased the freezing time in the context test 1, 3, and 7 days after surgery compared to the control condition (Fig. 2a–c; *p* < 0.05 for each). Postoperative JWH133 treatment reduced the freezing time in the context test on days 3 and 7 after surgery (Fig. 2a; *p* < 0.05), while postoperative AM630 treatment reduced the freezing time in the context test only 7 days after surgery (Fig. 2a–c; *p* < 0.05 for each), compared to the control condition. Furthermore, postoperative JWH133 treatment increased the freezing time in the context test on postoperative days 3 and 7 (Fig. 2b, c; *p* < 0.05 for both), while postoperative AM630 treatment reduced the freezing on day 7, compared to the surgery group (Fig. 2c; *p* < 0.05).

The tone test of the FCT was performed to assess hippocampal-independent memory, such as the prefrontal cortex. A repeated measures ANOVA for the data reported in Fig. 2 detected no significant difference among the groups in the freezing time in the tone test (Fig. 2d–f; overall *F* (6,35) = 0.253, *n* = 42, *p* = 0.955).

### Expression of proinflammatory factors in the hippocampus

**Fig. 3:**
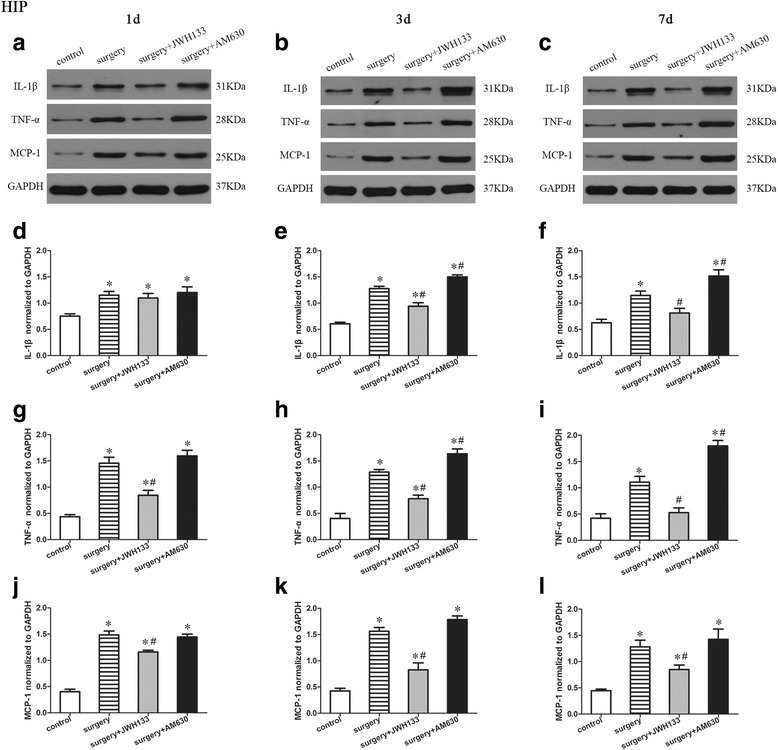
Surgery resulted in an increased expression of proinflammatory factors in the hippocampus. JWH133 treatment alleviated the surgery-induced upregulation of proinflammatory factors in the hippocampus, while AM630 aggravated it. Representative Western blotting bands show the expression of IL-1β, TNF-α, and MCP-1 in the hippocampus 1, 3, and 7 days after surgery (**a**–**c**). The expression of IL-1β, TNF-α, and MCP-1 were normalized to that of GAPDH as an internal control (**d**–**l**). The data are plotted as the mean ± standard error of the mean for each group (*n* = 3 per group). *p < 0.05 versus the control group, ^#^ *p* < 0.05 versus the surgery group

We determined the effects of surgery and postoperative JWH133/AM630 treatment on the expression of IL-1β, TNF-α, and MCP-1 in the hippocampus of the mice on postoperative days 1, 3, and 7 by Western blotting (Fig. 3). A repeated measures ANOVA for the data reported in Fig. 3 identified significant differences among groups in the hippocampal expression of IL-1β (Fig. 3d–f; overall *F* (3,8) = 26.420, *n* = 12, *p* < 0.05), TNF-α (Fig. 3g–i; overall *F* (3,8) = 88.152, *n* = 12, *p* < 0.05), and MCP-1 (Fig. 3j–l; overall *F* (3,8) = 90.259, *n* = 12, *p* < 0.05). Surgery induced a marked increase in the expression of IL-1β, TNF-α, and MCP-1 in the hippocampus on postoperative days 1, 3, and 7 compared to the control group (Fig. 3a–c). Compared to the mice in surgery group, those that received postoperative JWH133 treatment had decreased hippocampal IL-1β expression on postoperative days 3 and 7 (Fig. 3b, c, e, f; *p* < 0.05 for all), as well as decreased hippocampal expression of TNF-α (Fig. 3a–c, g–i; *p* < 0.05 for each) and MCP-1 (Fig. 3a–c, j–l; *p* < 0.05 for each) on postoperative days 1, 3, and 7. Postoperative AM630 treatment increased the hippocampal expression of IL-1β (Fig. 3b, c, e, f; *p* < 0.05 for all) and TNF-α (Fig. 3b, c, h, i; *p* < 0.05 for all) on postoperative days 3 and 7.

### Expression of proinflammatory factors in the prefrontal cortex

**Fig. 4:**
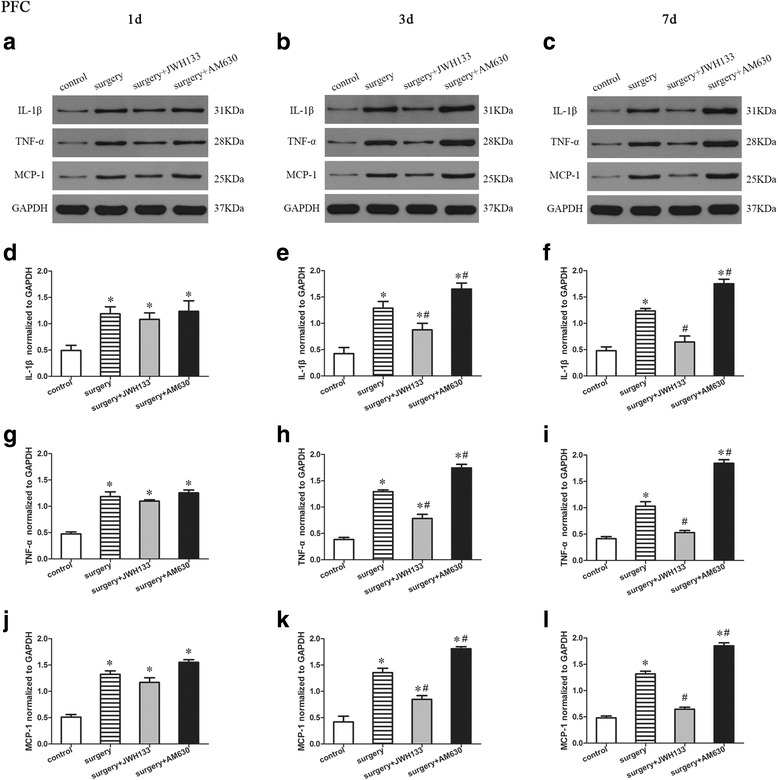
Surgery resulted in an increased expression of proinflammatory factors in the prefrontal cortex. JWH133 treatment alleviated the surgery-induced upregulation of proinflammatory factors in the prefrontal cortex, while AM630 aggravated it. Representative Western blotting bands show the expression of IL-1β, TNF-α, and MCP-1 in the prefrontal cortex 1, 3, and 7 days after surgery (**a**–**c**). Expression levels of IL-1β, TNF-α, and MCP-1 were normalized to that of GAPDH as an internal control (**d**–**l**). The data are plotted as the mean ± standard error of the mean for each group (*n* = 3 per group). *p < 0.05 versus the control group, ^#^ *p* < 0.05 versus the surgery group

We determined the effects of surgery and postoperative JWH133/AM630 treatment on the expression of IL-1β, TNF-α, and MCP-1 in the prefrontal cortex of the mice on postoperative days 1, 3, and 7 by Western blotting (Fig. 4). A repeated measures ANOVA for the data reported in Fig. 4 identified significant differences in the expression of IL-1β (Fig. 4d–f; overall *F* (3,8) = 30.251, *n* = 12, *p* < 0.05), TNF-α (Fig. 4g–i; overall *F* (3,8) = 225.153, *n* = 12, *p* < 0.05), and MCP-1 (Fig. 4j–l; overall *F* (3,8) = 105.022, *n* = 12, *p* < 0.05) in the prefrontal cortex among the groups. Surgery induced a marked increase in the expression of IL-1β, TNF-α, and MCP-1 in the prefrontal cortex 1, 3, and 7 days after surgery compared to the control group (Fig. 4a–c). Compared to the levels in the surgery group, the expression of IL-1β, TNF-α, and MCP-1 were decreased by postoperative JWH133 treatment and increased by postoperative AM630 treatment in the prefrontal cortex on days 3 and 7 postoperatively (Fig. 4b, c, e, f, h, i, k, l; *p* < 0.05 for each).

### CB2R expression in the hippocampus

**Fig. 5:**
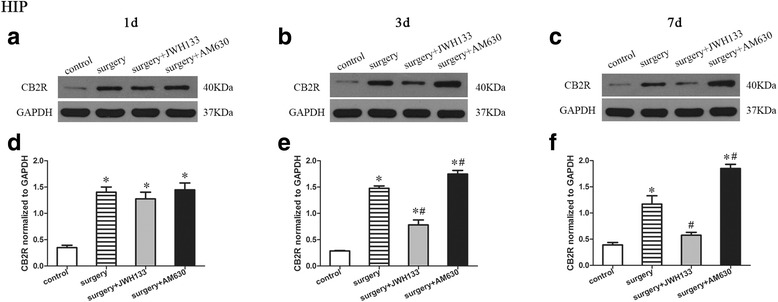
Surgery resulted in an increased expression of CB2R in the hippocampus. JWH133 treatment alleviated the surgery-induced upregulation of CB2R in the hippocampus, while AM630 aggravated it. Representative Western blotting bands show the expression of CB2R in the hippocampus 1, 3, and 7 days after surgery (**a**–**c**). The expression of CB2R was normalized to that of GAPDH as an internal control (**d**–**f**). The data are plotted as the mean ± standard error of the mean for each group (*n* = 3 per group). *p < 0.05 versus the control group, ^#^ *p* < 0.05 versus the surgery group

A repeated measures ANOVA for the data reported in Fig. 5 identified significant differences in CB2R expression (Fig. 5d–f; overall *F* (3,8) = 79.639, *n* = 12, *p* < 0.05) in the hippocampus among the groups. CB2R expression was enhanced in the hippocampus of the mice in the surgery group compared to the control group on postoperative days 1, 3, and 7 (Fig. 5). Postoperative daily administration of JWH133 resulted in reduced CB2R expression on postoperative days 3 and 7 (Fig. 5b, c, e, f; *p* < 0.05 for all), while postoperative daily administration of AM630 resulted in increased CB2R expression on postoperative days 3 and 7 (Fig. 5b, c, e, f; *p* < 0.05 for all) compared to the surgery group, in which mice were daily treated with vehicle. Interestingly, after postoperative JWH133 treatment for 7 days, the expression of CB2R in the hippocampus did not differ from that of the control group (Fig. 5c, f; *p* > 0.05).

### CB2R expression in the prefrontal cortex

**Fig. 6:**
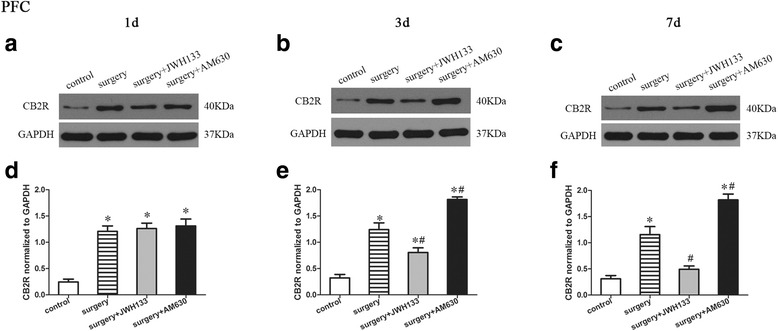
Surgery resulted in an increased expression of CB2R in the prefrontal cortex. JWH133 treatment alleviated the surgery-induced upregulation of CB2R in the prefrontal cortex, while AM630 aggravated it. Representative Western blotting bands show the expression of the CB2R in the prefrontal cortex 1, 3, and 7 days after surgery (**a**–**c**). The expression of CB2R was normalized to that of GAPDH as an internal control (**d**–**f**). The data are plotted as the mean ± standard error of the mean for each group (*n* = 3 per group). *p < 0.05 versus the control group, ^#^ *p* < 0.05 versus the surgery group

A repeated measures ANOVA for the data reported in Fig. 6 identified significant differences among the groups in CB2R expression in the prefrontal cortex (Fig. 6d–f; overall *F* (3,8) = 138.324, *n* = 12, *p* < 0.05). The expression of CB2R was enhanced in the prefrontal cortex of mice in the surgery group compared to the control group on postoperative days 1, 3, and 7 (Fig. 6). Daily postoperative administration of JWH133 to the surgical mice resulted in reduced CB2R expression on postoperative days 3 and 7 (Fig. 6b, c, e, f; *p* < 0.05 for all), while daily postoperative administration of AM630 to surgical mice resulted in increased CB2R expression on postoperative days 3 and 7 (Fig. 6b, c, e, f; *p* < 0.05 for all), compared to the vehicle-treated surgical mice. Interestingly, after postoperative JWH133 treatment for 7 days, there was no significant difference between the surgery + JWH133 and control groups in the expression of CB2R in the prefrontal cortex (Fig. 6c, f; *p* > 0.05).

### CD11b expression in the CA1 area of the hippocampus

**Fig. 7:**
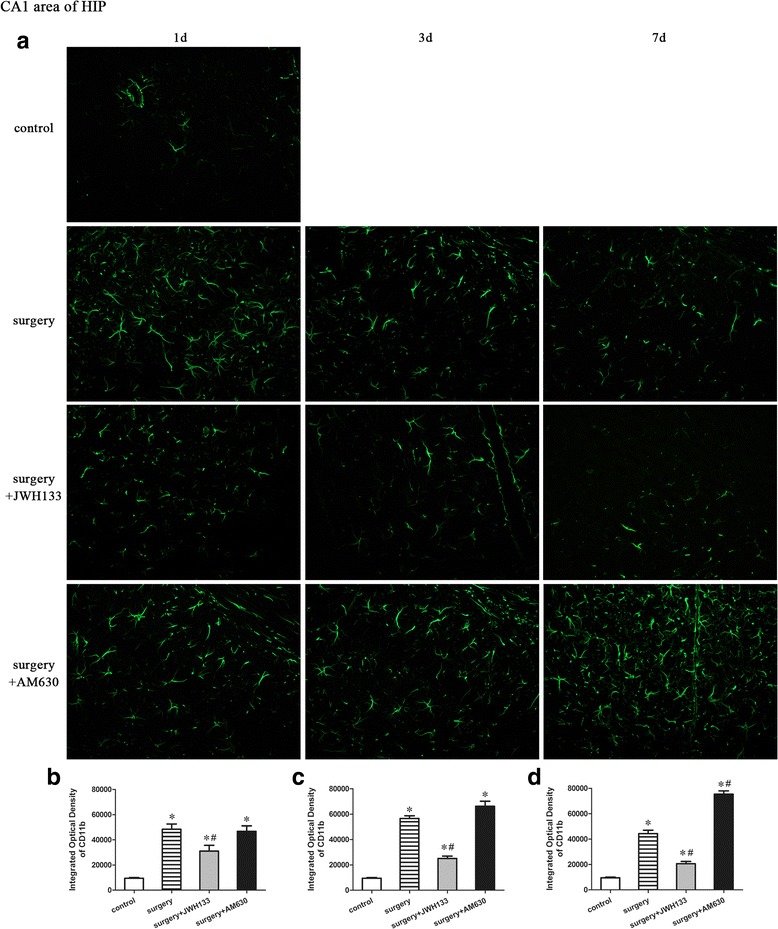
Surgery resulted in an increased expression of CD11b in the CA1 area of the hippocampus. JWH133 treatment alleviated the surgery-induced upregulation of CD11b in the CA1 area of the hippocampus, while AM630 aggravated it. Representative immunofluorescence images show the expression of CD11b (*green pixels*) in the hippocampal CA1 area of mice 1, 3, and 7 days after surgery (**a**). Original magnification = ×200. Quantitative analyses of the immunofluorescence images (**b**–**d**). The data are plotted as the mean ± standard error of the mean for each group (*n* = 3 per group). *p < 0.05 versus the control group, ^#^ *p* < 0.05 versus the surgery group

A repeated measures ANOVA for the data reported in Fig. 7 identified significant differences among the groups in CD11b expression in the CA1 area of the hippocampus (Fig. 7b–d; overall *F* (3,20) = 217.728, *n* = 24, *p* < 0.05). The expression of microglial marker CD11b was upregulated in the CA1 area of the hippocampus of mice in the surgery group on postoperative days 1, 3, and 7 compared to the control group (Fig. 7). Postoperative daily administration of JWH133 resulted in reduced CD11b expression on postoperative days 1, 3, and 7 (Fig. 7a–d; *p* < 0.05 for each), while postoperative daily administration of AM630 resulted in increased CD11b expression on postoperative day 7 (Fig. 7a, d; *p* < 0.05), compared to the surgery group, which received daily vehicle treatment.

### CD11b expression in the medial prefrontal cortex

**Fig. 8:**
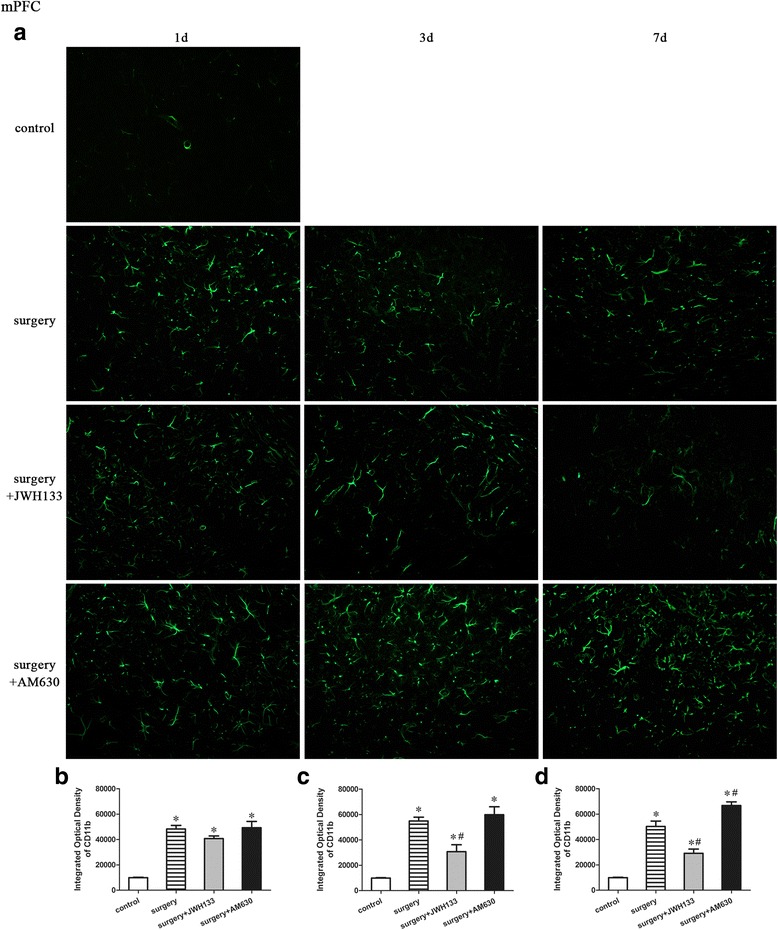
Surgery resulted in an increased expression of CD11b in the mPFC. JWH133 treatment alleviated the surgery-induced upregulation of CD11b in mPFC, while AM630 aggravated it. Representative immunofluorescence images show the expression of CD11b (*green pixels*) in the mPFC of mice 1, 3, and 7 days after surgery (**a**). Original magnification = ×200. Quantitative analyses of the immunofluorescence images (**b**–**d**). The data are plotted as the mean ± standard error of the mean for each group (*n* = 3 per group). *p < 0.05 versus the control group, ^#^ *p* < 0.05 versus the surgery group

A repeated measures ANOVA for the data reported in Fig. 8 identified significant differences in CD11b expression (Fig. 8b–d; overall *F* (3,20) = 139.834, *n* = 24, *p* < 0.05) in the medial prefrontal cortex (mPFC) among the groups. The expression of microglial marker CD11b was upregulated in the medial prefrontal cortex of the mice in the surgery group on postoperative days 1, 3, and 7 compared to the control group (Fig. 8). Postoperative daily administration of JWH133 to surgical mice resulted in reduced CD11b expression on postoperative days 3 and 7 (Fig. 8a, c, d; *p* < 0.05 for all), while postoperative daily administration of AM630 resulted in increased CD11b expression on postoperative day 7 (Fig. 8a, d; *p* < 0.05) compared to the surgery group, which received daily vehicle treatment.

## Discussion

The aim of the current study was to assess the associations among cognitive impairment, neuroinflammation, and CB2R expression in adult mice subjected to orthopedic surgery under isoflurane anesthesia. To our knowledge, this is the first evaluation of the influence of selective CB2R ligands on learning and memory in surgical mice. In addition, we explored the effects of pharmacological activation or blockade of CB2R by systemic administration of the CB2R agonist JWH133 or the CB2R antagonist AM630, respectively, on learning and memory in surgical mice. We also assessed the inflammatory responses in brain regions closely related to memory.

Our results showed that hippocampal-dependent memory was impaired following surgery. Postoperative JWH133 treatment attenuated the surgery-induced memory loss, while postoperative AM630 treatment aggravated the surgery-induced memory loss. With respect to neuroinflammatory responses in memory-related brain regions, the present study showed increased IL-1β, TNF-α, and MCP-1 expression in the hippocampus and prefrontal cortex following surgery. Postoperative JWH133 treatment reversed the increased expression of IL-1β, TNF-α, and MCP-1 in these two brain regions; in contrast, postoperative AM630 treatment enhanced the increased expression of IL-1β, TNF-α, and MCP-1 in these two brain regions. Moreover, our study showed an upregulation of CB2R in the hippocampus and prefrontal cortex of mice in the surgery group on postoperative day 1, but daily administration of JWH133 reversed the expression of CB2R in both brain regions to the level of the control group on postoperative day 7. Furthermore, we found an upregulation of CD11b in hippocampal area CA1 and the mPFC of surgical mice and a relative downregulation by postoperative JWH133 treatment.

Neuroinflammation has been associated with cognitive impairment and stands out among the mechanisms underlying POCD [25]. The cytokines IL-1β and TNF-α are widely described neuroinflammatory mediators associated with cognitive impairment [4, 26, 27]. Moreover, recent studies have revealed an increased expression of MCP-1 mRNA in the hippocampus of mice with POCD [28, 29]. An impaired memory paralleled by increased IL-1β, TNF-α, and MCP-1 concentrations in the hippocampus and prefrontal cortex following surgery was observed in the present study, which agrees with the above studies indicating a co-occurrence of neuroinflammation and postoperative cognitive impairment.

Our study focused on the effects of selective CB2R ligands on postoperative cognition in adult mice. The finding that postoperative treatment with the CB2R agonist JWH133 ameliorated postoperative memory loss and attenuated the increased levels of proinflammatory factors in the hippocampus and prefrontal cortex indicates that the activation of CB2R may have a protective effect on early postoperative cognition. Indeed, the protective effect of CB2R agonists has been demonstrated in other animal models and behavioral assessments [14–17]. For example, a previous study revealed that MDA7, another CB2R agonist, could ameliorate the memory deficiency in the Morris water maze test induced by bilateral microinjection of amyloid-β into the hippocampal CA1 area of rats [14]. In another study, JWH133 was found to enhance aversive memory consolidation in the step-down inhibitory avoidance test, whereas AM630 impaired it [23]. Similarly, the CB2R agonist O-1966 was demonstrated to reverse the retention deficit in a food-motivated operant learning task in a stroke model [15]. Neuropathological findings in human brains suggest that the upregulation of CB2R is a common response against different types of chronic injury in the human CNS, and the selective presence of CB2R in microglial cells strongly suggests an important role for this receptor in disease-associated neuroinflammatory processes [9]. The current finding that CB2R expression was upregulated in the hippocampus and prefrontal cortex of mice 1 day after surgery but was reversed by postoperative JWH133 treatment to the level of the control group 7 days after surgery, adding support for the notion that CB2R acts as a negative feedback regulator [14]. We speculate that the neuroinflammatory response may initiate the upregulation of CB2R, whose subsequent activation limits the extent of the neuroinflammatory response, leading to its final downregulation. Taken together, these results support the notion that the neuroprotective effects of CB2R activation are mediated by its suppression of microglial activation and proinflammatory cytokine production [19].

Interestingly, although postoperative JWH133 treatment significantly decreased TNF-α and MCP-1 expression 1 day post surgery in the hippocampus, it failed to do so in the PFC. Similarly, the effects of JWH133 administration on CD11b expression also differed between the hippocampus and PFC. These results were not surprising, given that various brain areas may have different mechanisms to regulate neuroinflammation caused by surgical trauma, although the precise mechanisms have yet to be identified [30, 31]. For example, in rodents, a peripheral injection of lipopolysaccharide (LPS) was previously shown to produce an acute inflammatory response in which IL-1β levels are elevated in the hippocampus but not in the PFC [30, 31]. In addition, the time course of CB2R expression after surgical insults, the interactions between CB2R and CB1R, and the exact pathways of CB2R-mediated inflammation among different brain regions are still largely unknown. Thus, future studies are necessary to investigate the time course and regulatory mechanisms of CB2R activation across different brain areas in surgery-induced neuroinflammation.

It should be noted that the mice exposed to anesthesia alone exhibited no memory deficit, which concurs with previous studies suggesting that anesthesia alone may have no significant influence on POCD development [4, 26, 27]. However, other studies [32–34] have suggested that the anesthetics isoflurane and sevoflurane induce learning and memory impairment. This discrepancy may be attributed primarily to the following two differences among studies: first, the anesthesia strategy, which is the concentration of isoflurane and the duration exposure; and second, the age of the experimental animals, which has been identified as the main predisposing factor for postoperative cognitive dysfunction. In addition, the surgical mice presented a decreased freezing time in the context test, but no significant difference in freezing time in the tone test, compared with that in the control group. In other words, surgery impaired the hippocampal-dependent but not hippocampal-independent memory in mice, which coincides with the finding that hippocampal-dependent learning and memory is specifically vulnerable to surgery-induced impairment in young adult rats [26].

Furthermore, although increased neuroinflammation was also found in the PFC following surgery, hippocampal-dependent but not hippocampal-independent functions were impaired in our mice. This outcome was not surprising. On the one hand, as mentioned previously, hippocampal-dependent learning and memory is especially vulnerable to inflammatory insults [7]; on the other hand, dysfunction in particular cognitive domains occurs only when specific underlying intra-neuronal pathways, such as the brain-derived neurotrophic factor (BDNF)-mediated pathway, are significantly affected [26]. The BDNF pathway, in particular, has been implicated as a mediator between neuroinflammation and cognitive impairment [7]. It is possible that BDNF levels in the PFC remained intact, as did the cognitive functions mediated by this brain region.

In the current study, the dose and administration time of JWH133 and AM630 were selected based on previous studies [21–23] to ensure adequate functionality. However, our results demonstrated no effects of the CB2 agonist/antagonist on the performance of mice in behavioral tests on postoperative day 1. These results were inconsistent with those of a previous study [23], which indicated that JWH133 and AM630 had opposing effects on the performance of mice in the step-down inhibitory avoidance test in the early postsurgical timeframe (1 and 24 h after training). The discrepant findings concerning CB2 receptor ligands are attributable to differences in the treatment duration, experimental conditions, animal model, strain, and species [35]. For example, García-Gutiérrez et al. [21] reported that JWH133 had no effect in the light/dark box test after acute treatment and elicited an anxiogenic response after chronic treatment. Likewise, AM630 can induce anxiogenic and anxiolytic activity in mice after acute and chronic treatment, respectively.

The current study has some limitations. First, we did not employ CB1R-selective ligands or non-selective ligands of cannabinoid receptors, so we cannot exclude possible interactions of the cannabinoid receptors. Additionally, endogenous ligands (endocannabinoids) have been demonstrated to play a key neuromodulatory role in the central nervous system [36]. The best-characterized endocannabinoids are arachidonoylethanolamide (AEA), also known as anandamide [37], and 2-arachidonoylglycerol (2-AG) [38]. AEA and 2-AG behave as a partial and full agonist, respectively, at both cannabinoid receptors (CB1R and CB2R) [39, 40]. AEA has been reported to play an important role in the modulation of memory consolidation and in anxiety-like responses [41]. In the current study, we did not measure the levels of AEA and 2-AG in the brain. Future investigations should examine the potential effects of these endocannabinoids on the surgery-induced neuroinflammation and cognitive impairments. Second, our study emphasized the surgery-induced neuroinflammation in the hippocampus and prefrontal cortex of mice, but systemic inflammation was not examined. Moreover, the synaptic plasticity alterations and the neuronal pathways that may have contributed more directly to cognitive function were not investigated. Third, in addition to memory, CB2R may be involved in the regulation of anxiety. JWH133 treatment was previously shown to increase anxiety-like behavior, whereas AM630 has anxiogenic effects [21]; thus, we cannot rule out the possibility that an alteration in anxiety levels by CB2R ligands influenced the freezing behavior in the fear conditioning test in mice. Fourth, in the current study, we just used local analgesics, but not other analgesics such as buprenorphine, to control incisional pain. These local anesthetics may not be effective for nociceptive processes from the fracture and its healing callus. Additionally, CB2R activation has been reported to attenuate nociceptive responses in models of neuropathic and osteoarthritic pain [42, 43]. More importantly, in the present study, we did not perform any pain-related behavioral tests. Therefore, postoperative pain could be a confounding factor in the present study because incisional pain induces cognitive impairment in rodents [44]. Fifth, because the present study lacked CB2R knockout mice or a surgery + antagonist + agonist group (surgery + AM630 + JWH133 group), we cannot demonstrate unequivocally the contribution of CB2R to surgery-induced neuroinflammation and cognitive impairment. Finally, because it is difficult and expensive to use aged mice, we used only adult mice in our experiment. Similarly, we only used male mice in the experiments, because they were intended to be part of a pilot study. These limitations indicate the need for further investigations.

## Conclusions

In summary, the results of the current study show that CB2R activation has a potential protective effect on memory in the early postoperative stage. Treatment with the CB2R agonist JWH133 dampened neuroinflammation and enhanced memory following surgery, whereas treatment with the CB2R antagonist AM630 aggravated neuroinflammation and worsened memory following surgery. Taken together, these results highlight CB2R as a potential target for the treatment of postoperative cognitive dysfunction, pending further investigations.

## Additional file

Additional file 1: Figure S1. All mice groups showed no significant difference in freezing time of training phase of fear conditioning (one-way ANOVA, *F* = 8.564, *n* = 42, *p* = 0.110). Data are plotted as mean ± standard error of the mean for each group (*n* = 6 per group). (PDF 143 kb)

## Abbreviations

ANOVA: Analysis of variance
CB1R: Cannabinoid receptor type 1
CB2R: Cannabinoid receptor type 2
CD11b: Cluster of differentiation 11b
DMSO: Dimethyl sulfoxide
EDTA: Ethylenediamine tetra-acetic acid
FCT: Fear conditioning test
GAPDH: Glyceraldehyde-3-phosphate dehydrogenase
HIP: Hippocampus
IL-1β: Interleukin-1 beta
MCP-1: Monocyte chemotactic protein 1
mPFC: Medial prefrontal cortex
PBS: Phosphate buffer saline
POCD: Postoperative cognitive dysfunction
SEM: Standard error of the mean
TNF-α: Tumor necrosis factor-alpha
