# Supplementary material for: Activation of cannabinoid receptor type 2 attenuates surgery-induced cognitive impairment in mice through anti-inflammatory activity
Source: J Neuroinflammation. 2017 Jul 19;14:138. doi: 10.1186/s12974-017-0913-7 (PMC5518095; doi:10.1186/s12974-017-0913-7)
Supplement: Additional file 1: Figure S1. — All mice groups showed no significant difference in freezing time of training phase of fear conditioning (one-way ANOVA, F = 8.564, n = 42, p = 0.110). Data are plotted as mean ± standard error of the mean for each group (n = 6 per group). (PDF 143 kb) [file 12974_2017_913_MOESM1_ESM.pdf]

Performance of mice in training phase of fear conditioning

The freezing time in training phase of fear conditioning showed no significant difference among the groups (Figure. one-way ANOVA, F=8.564, n=42, p=0.110), indicating the equivalent baseline of learning and memory of mice groups.

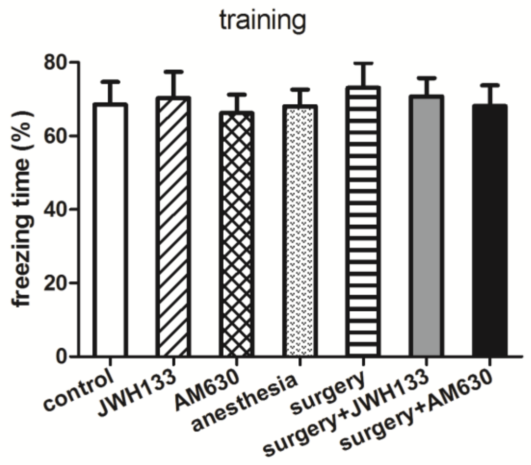

Figure. All mice groups showed no significant difference in freezing time of training phase of fear conditioning**.** Data are plotted as mean ± standard error of the mean for each group (n=6 per group).
